# Socio‐Ecological Factors Influencing Maternal and Child Health Outcomes During Floods in South Punjab Pakistan 2025: A Mixed‐Methods Approach

**DOI:** 10.1111/hex.70624

**Published:** 2026-02-26

**Authors:** Muhammad Muneeb Hassan, Badr S. Alnssyan, Muhammad Aman Ullah, Muhammad Ameeq, Alpha Kargbo

**Affiliations:** ^1^ Department of Statistics DHQ Hospital Muzaffargarh Muzaffargarh Punjab Pakistan; ^2^ Faculty of Business Multimedia University, Jalan Ayer Keroh Lama Melaka Malaysia; ^3^ Department of Management Information Systems College of Business and Economics, Qassim University Buraydah Saudi Arabia; ^4^ Department of Statistics Bahauddin Zakariya University Multan Punjab Pakistan; ^5^ Department of Statistics GHSS Lab A/W (PECTAA) Muzaffargarh Punjab Pakistan; ^6^ Department of Physical and Natural Sciences University of The Gambia Serrekunda Gambia

**Keywords:** cultural perception, infertility, reproductive health, statistical model and socioeconomic status

## Abstract

**Background:**

South Punjab, Pakistan, is located approximately nearby the Indus and Chanab rivers. Heavy monsoon and climate change make the situation worse every year, and these areas suffer from severe flooding. Millions of people face challenges in accessing the medical facility, hindered resource availability, damaged infrastructure and a potentially increased socio‐ecological problem.

**Objectives:**

The prime objective of the study was to examine socio‐ecological factors, hurdles to access healthcare facilities, and family coping methods that impact the child's and mother's health during the flood.

**Methods:**

We used the mixed‐method approach, including a quantitative survey of 560 women aged 18–49 that lived in the flood‐affected region (Bahawalpur, Multan and Muzaffargarh) and thematic analysis of 42 participants with in‐depth interviews performed by using NVivo software. Binary logistic regression was used to analyse predictors of maternal healthcare challenges. All statistical analyses were performed using SPSS‐26 and R‐Studio.

**Results:**

Primary conclusive challenges to access to maternal healthcare are limited access to clean water (OR = 3.239, CI: 1.494–6.561, *p* = 0.01), food insecurity (OR = 3.239, CI: 1.378–7.604, *p* = 0.01) and displacement (OR = 2.792, CI: 1.798–6.013, *p* = 0.01). Qualitative themes highlight dependence on informal networks, environmental pollution, and institutional overload.

**Conclusion:**

Water scarcity, food insecurity, and displacement significantly affected maternal and child health during the 2025 flood. Nutritional supplies, mobile healthcare and community training must be prioritised. Equitable health policies must be supported by future research into longitudinal recovery.

## Patient or Public Contribution

This mixed‐methods study examined the socio‐ecological factors affecting maternal and child health during the 2025 Punjab floods in Pakistan. This proves the statistical validity and illustrates diverse scenarios with quantitative and qualitative data. We learnt that health and environmental issues intersect. The quantitative survey began with 597 participants, 18–49‐year‐old women from flood‐ravaged South Punjab (Multan, Bahawalpur and Muzaffargarh). After eliminating 37 incomplete or inaccurate responses, 560 respondents remained. Their contribution is necessary for shaping the study design. Eight of the 50 participants in the qualitative phase declined or dropped out of the study. The final qualitative sample included 42 hospitals, public/private relief camps, roadsides, and school respondents. A qualitative study is used for thematic analysis to reach out to the reason behind the family being displaced from their homes. They strengthened the case for maternal and child health recommendations and helped identify flood victims.

## 1 Introduction

In the world, floods are among the most frequent and deadly natural disasters [1]. It has a bigger impact on people with few resources in developing areas because it forces people to move, destroys infrastructure, and raises public health concerns [2]. Pakistan has various types of land and extensive river areas, with a climate that relies on monsoons [3]. The situation becomes worse when the climate changes; that becomes the reason for flooding. Since the 20th century, Pakistan has faced terrible flooding every year due to heavy monsoons [4, 5]. After the 1950 super flood, which was caused by heavy monsoon rains and killed over 2000 people and displaced 10 million from their homes in Sindh and Punjab, Pakistan has experienced numerous disasters [6, 7]. Flooding occurred repeatedly in the south Punjab region in 1955–1959, causing rivers to overflow and poor drainage, which killed hundreds of people and destroyed numerous crops. An unprecedented number of monsoons in 2010 led to devastating floods that impacted twenty million people nationwide. They killed 2000 people and caused $10 billion in damage. These disasters have happened frequently, putting a lot of stress on healthcare systems. They have especially affected the health of mothers and children by making it hard to access services, spreading diseases, and making food challenging to find [6, 7, 8, 9]. The floods in Pakistan in 2025, which started with heavy rain before the monsoon season and lasted through the monsoon season, were the worst ever in Punjab province and continually became worse [10, 11, 12]. The floods caused flash floods, landslides, and rivers to overflow, especially the Indus, Jhelum, Chenab, Ravi, and Sutlej Rivers [13, 14]. Nearly 1000 people had perished nationwide by the end of August. A million people were impacted, 4200 homes were destroyed, and crops in the country's breadbasket region were severely damaged [10, 15]. In Punjab, which experienced the most severe flooding in its history, approximately two million individuals were impacted, with nearly one million compelled to evacuate their residences, including children and families with young children. In many cities of the districts (Multan, Muzaffargarh and Bahawalpur), 1400 villages experienced saturation due to compromised infrastructure. Environmental changes that affect social factors can worsen various mother and child health problems, including disrupted prenatal care [16], infectious diseases [17, 18, 19, 20] and nutritional deficiencies [21].

Our research carried out the socio ecological model [21, 22, 23] to investigate the impact of different child and mother helth factor during the 2025 flood [24]. These factors cover personal variables such as age and socioeconomic status, as well as community resources and environmental conditions [23]. If we compare the previous studies that have identified correlation between insufficient education, low household income, and environmental challenges like inadequate sanitation and water scarcity, resulting in adverse outcomes, including reduced access to prenatal and paediatric care [16, 25, 26, 27, 28]. This research employed a mixed‐methods approach [24, 29, 30], integrating quantitative survey data from flood‐impacted residences with in‐depth interviews regarding coping strategies to explain these dynamics and inform resilience‐building efforts. Previously, the researchers only explored rural‐urban disparities in demographic and marital status, but they overlooked the presence of mothers and child care.

The study aims to examine the influence of socio‐ecological factors on maternal and child health outcomes during the floods situation. It identifies barriers to accessing healthcare and essential resources for families and analyses family survival strategies. We believe that families with lower socioeconomic status and greater environmental stressors, such as inconsistent access to clean water and food scarcity, will encounter greater difficulties in obtaining maternal and paediatric care. In comparison to families with more protective factors, our findings will lead to less favourable health results.

## 2 Methods

This mixed‐methods study investigated the socio‐ecological determinants influencing maternal and child health during the 2025 Punjab floods in Pakistan. It employs both quantitative and qualitative data to establish statistical validity and illustrate diverse scenarios. We have learned how health and environmental problems affect each other. The quantitative survey began with 597 women from flood‐affected South Punjab (Multan, Bahawalpur and Muzaffargarh), aged 18–49. After eliminating 37 respondents who did not provide complete or precise responses, 560 were left to respond. Eight of the 50 people who were asked to participate in the qualitative phase dropped out or said they did not want to participate. Trained enumerators manually recorded responses in diary notes to ensure field accuracy while collecting data in person using a validated, pre‐tested paper questionnaire. The tool's content and face validity were assessed by biostatisticians and maternal health specialists. After its initial development in English, the questionnaire underwent a forward‐backward translation procedure before being translated into Urdu. Both the socio‐ecological factors (alpha = 0.80) and the maternal healthcare access items (alpha = 0.83), when tested for internal consistency reliability using Cronbach's alpha, demonstrated good reliability.

The final qualitative sample included 42 participants. We acquired this information about flood‐affected families’ initial experiences using NVivo for thematic analysis from public/private hospitals and relief camp respondents, including roadside and school facilities. Sequential descriptions are used. The quantitative results were supported by qualitative data collection before the qualitative analysis. This method was used in a disaster framework to address flooding's many challenges, difficult access points, and complex narratives of experiences and coping mechanisms. Overcrowded hospitals and informal camp networks were major themes. In District Headquarter Hospital Muzaffargarh interviewed people from the start. Second, a common theme in reports from schools and temporary housing was that families who had been affected by the disaster relied on informal networks for assistance. As part of this practice, people would share food, water, and home remedies, such as herbal remedies for fever and diarrhoea. Receiving capability of connecting was important for survival, but it was not always enough to meet the huge medical needs, especially when aid deliveries were late.

### 2.1 Participants and Study Setting

The study was done in South Punjab's Multan, Bahawalpur and Muzaffargarh, which had been affected by floods. The Provincial Disaster Management Authority (PDMA) said that there was much of flooding that forced people to leave their homes. They are in both cities and the country. Since it was close to the flooded Indus and Chenab rivers, Muzaffargarh suffered most severely in 2025. Here is the place where the five rivers of Pakistan meet. Only women aged 18–49 who lived in areas affected by the flood, had at least one child under five, and had direct flood damage were eligible. Purposeful sampling ensured diversity across socioeconomic strata, residential categories (rural and urban), and displacement conditions (temporary, permanent or non‐displaced).

### 2.2 Data Analysis

Sample attributes and variable distributions are described by descriptive statistics including frequencies, percentages, means, and standard deviations. Chi‐square tests were used to examine categorical variables (rural residence and clean water access) and binary logistic regression to predict adverse health outcomes (very difficult maternal care access). We added independent variables like income, displacement, and sanitation one at a time, accounting for age and education that could affect results.

We verified the model's assumptions, including multicollinearity (variance inflation factor < 5) and goodness‐of‐fit (*p* > 0.05 on the Hosmer‐Lemeshow test). The significance level was set at *p* < 0.05. Mixed‐methods integration occurred through joint display matrices, where qualitative themes clarified quantitative patterns, specifically the correlation between low‐income groups and increased food insecurity linked to disrupted camp distributions. We utilised R and SPSS version 26 for the quantitative analysis.

## 3 Results

The results of the mixed‐method approach explain the socioeconomic factors affecting maternal and child health during the flood in Pakistan. Our study combines both quantitative and qualitative survey data from 560 women aged 18–49 from the flood‐affected area. The result shows that the large variation in health caused the environmental stress, socioeconomic variation and systematic challenge. These differences are most prominent in the low‐income, rural areas and displaced houses. The results of binary logistic regression revealed that access to clean water, food security and displacement were highly influential variables that produce challenges in accessing maternal healthcare.

### 3.1 Descriptive Statistics of Socio‐Demographic and Environmental Factors

Women aged 26–35 years (*n* = 256, 46%), aged 18–25 (*n* = 143, 25%) and aged 36–45 (26%, *n* = 144). Most of them (*n* = 348, 62%) live in rural areas, and only (*n* = 212, 38%) live in urban areas, showing the region divide between the rural and urban. Socioeconomic challenges were clear, with 60.2% (*n* = 337) of households reporting monthly incomes above 30,000 PKR, 6.8% (*n* = 38) below 10,000, 14.1% (*n* = 79) between 10,000–20,000, and 18.9% (*n* = 106) between 20,001–30,000. The categories of education that were very low were no formal education (*n* = 285, 52%) and secondary or higher education (*n* = 49, 8%). Most of them had at least one child under the age of 5. A total of 50.2% (*n* = 281) had 3–4 children, 36.2% (*n* = 205) had 1–2, and 13.2% (*n* = 74) had 5 or more. A large number of people were forced to leave their homes, with a significant number of people still residing in temporary quarters, a permanent quarter, and a small percentage remaining in their homes. A total of 36% of women (*n* = 202) were pregnant when the floods occurred; 6% (*n* = 34) gave birth during or after the floods; and 58% (*n* = 324) were not pregnant, highlighting the impact on maternal health. Several people found it difficult to get maternal healthcare; 39% (*n* = 218) said it was somewhat hard, 47% (*n* = 265) said it was neutral, and 7% (*n* = 42) said it was somewhat easy.

Access to child healthcare was even more limited: 81.4% (*n* = 456) found it somewhat difficult, 11.4% (*n* = 64) neutral, and only 4.3% (*n* = 24) somewhat easy or 2.9% (*n* = 16) very easy. Socio‐ecological factors emphasised environmental disruptions: access to clean water was occasionally available for 48.6% (*n* = 272), but infrequently (2.7%, *n* = 15) or never (2.1%, *n* = 12) for others, with only 10.4% (*n* = 58) having consistent access. Food security was also unstable, with 47% (*n* = 263) saying they sometimes had enough food, 3.8% (*n* = 21) saying they rarely did, and 0.5% (*n* = 3) saying they never did. 21.8% (*n* = 122) always had enough food. Most people (57%, *n* = 319) said the sanitation conditions were bad, and 38.3% (*n* = 214) said they were fair. Only 4.8% (*n* = 27) said they were very good. Not receiving aid, such as food or medical aid, worsened the vulnerabilities of 61% (*n* = 343) of respondents. Despite the lack of specific frequency information in the quantitative data, coping strategies included relying on family or community support, using home remedies, seeking out limited healthcare services, and temporarily relocating as mention in Table 1.

**Table 1:** Descriptive statistics of socio‐demographic and environmental factors among flood‐affected households in South Punjab, Pakistan, 2025.

Characteristics	Total (%)	Characteristics	Total (%)
**Section 1:** Demographic and Socioeconomic Information		**Section 3:** Child Health During Floods	
Age	—	Access to pediatric healthcare	**—**
18–25	143 (25.5)	Very easy	16 (2.9)
26–35	256 (45.7)	Somewhat easy	24 (4.3)
36–45	144 (25.7)	Neutral	64 (11.4)
15–49	17 (3)	Somewhat difficult	456 (81.4)
Area of residence	**—**	**Section 4:** Socio‐Ecological Factors	
Rural	348 (62.1)		
Urban	212 (37.9)		
Household monthly income (PKR)	**—**	Access to clean water during floods	**—**
Below 10,000	38 (6.8)	Always	58 (10.4)
10,000–20,000	79 (14.1)	Often	203 (36.3)
20,001–30,000	106 (18.9)	Sometimes	272 (48.6)
Above 30,000	337 (60.2)	Rarely	15 (2.7)
		Never	12 (2.1)
Education level	**—**	Food security during floods	**—**
No formal education	285 (50.9)	Always	122 (21.8)
Primary (up to Grade 5)	226 (40.4)	Often	151 (27)
Secondary or higher	49 (8.8)	Sometimes	263 (47)
		Rarely	21 (3.8)
		Never	3 (0.5)
Children under 5 years	**—**	Sanitation conditions	**—**
1–2	205 (36.2)	Fair	214 (38.3)
3–4	281 (50.2)	Poor	319 (57)
5 or more	74 (13.2)	Very good	27 (4.8)
Displacement due to floods	—	Receive support (e.g., food, medical aid) during the floods	—
Yes, temporarily displaced	392 (70)	Yes	**217 (38.7)**
Yes, permanently displaced	147 (26.3)	No	**343 (61.2)**
No, not displaced	21 (3.8)		
**Section 2:** Maternal Health During Flood		**Section 5:** Coping Mechanisms and Recommendations	
Pregnancy status during flood	—	What strategies did your family use to cope with health challenges during the 2025 floods?	—
Pregnant during flood	202 (36.1)	Relied on family/community support	122 (21.8)
Gave birth during/after flood	34 (6.1)	Used home remedies	151 (27)
Not pregnant	324 (57.9)	Sought healthcare services	263 (47)
		Relocated temporarily	24 (4.3)
Access to maternal healthcare during flood			
Very easy	42 (7.5)		
Somewhat easy	265 (47.3)		
Neutral	35 (6.3)		
Somewhat difficult	218 (38.9)		

*Note:* This table uses bold text only to differentiate main section headings (such as Section 1: Demographic and Socioeconomic Information) and primary category labels. This makes the multi‐section descriptive statistics for socio‐demographic, maternal, child health, and environmental factors easier to read and understand within the organizational structure.

**Figure 1:**
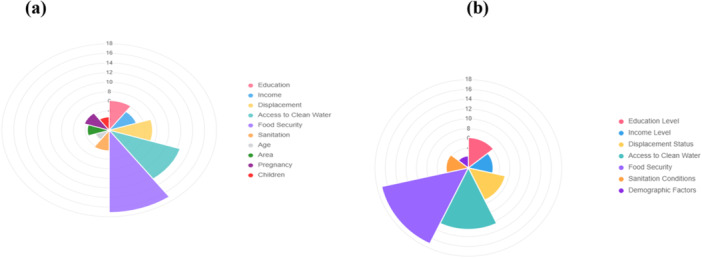
Distribution of access challenges and socio‐ecological stressors.

Access to maternal and child healthcare is visualised in the distribution in Figure 1a, which shows a prominent skew towards difficulty. Respondents found paediatric healthcare somewhat difficult (81%), and 39% reported maternal healthcare as somewhat difficult. This shows the widespread barriers to healthcare post‐flood. In Figure 1b, socioeconomic factor stressors described that access to clean water is available 49% and rarely or never is 5%, and food security is somewhat secure 47% and rarely or never is 4%. Sanitation conditions were very poor; 57% say further it highlights the environmental vulnerability and health risks for both mother and child, such as waterborne diseases and malnutrition.

**Figure 2:**
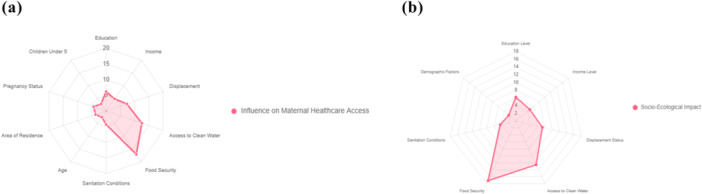
Predictors of maternal healthcare access difficulties.

The bar chart displays the odds ratios from the logistic regression, highlighting food security, rural residence and access to clean water as the most prominent factors, followed by displacement and lack of formal education, as illustrated in Figure 2a. These graphs show the strong influence of maternal and child healthcare. Food security, clean water, displacement and no formal education confirm the statistical reliability and the relative impact factor at a 95% confidence interval as shown in Figure 2b. These figures prominently show that food insecurity, environmental stressors, lack of clean water and health disparities align with the study objectives.

### 3.2 Bivariate and Regression Analyses

Bivariate analyses using Chi‐square tests established significant associations between important variables, such as living in a rural area and the difficulty of accessing clean water, confirming that geographic isolation increased environmental barriers. There was no multicollinearity and the model fit was good because the Hosmer‐Lemeshow *p*‐value was greater than 0.05. Both *p*‐value and variance inflation factor were below the 0.05 cutoff. Regression analysis showed a number of factors that significantly correlated with mothers’ problems to access healthcare.

Specifically, compared to incomes above 30,000 PKR, odds were 2.492 times higher for incomes below 10,000 PKR, 2.041 times higher for incomes between 10,000 and 20,000 PKR, and 1.380 times higher for incomes between 20,001 and 30,000 PKR. Because of the disruption to healthcare infrastructure and mobility restrictions, displacement was a significant barrier, increasing the odds by 2.792 times (95% CI: 1.798–6.013, OR = 2.792, *p* = 0.001). An extremely influential environmental factor was the availability of clean water, which increased the likelihood of difficulty by 3.239 times (95% CI: 1.494–6.561, OR = 3.239, *p* = 0.001), most likely as a result of the increased disease prevalence associated with contamination risks. An increased risk of difficulty of 3.835 times (95% CI: 1.378–7.604, OR = 3.835, *p* = 0.001), associated with food insecurity, contributed directly to malnutrition and impaired immunity in both mothers and their children. A 2.592 times increase in the odds of infectious disease was associated with unsanitary conditions (95% CI: 1.967–5.362, OR = 2.592, *p* = 0.002). Additional factors included rural residence (95% CI: 1.769–7.689, OR = 3.687, *p* = 0.001), pregnancy during the flood (95% CI: 1.994–8.654, OR = 3.387, *p* = 0.001), giving birth during or after the flood (95% CI: 1.914–9.307 OR = 4.241, *p* = 0.001), and having 3–4 children under 5 years (95% CI: 1.408–5.496, OR = 2.782, *p* = 0.003) or 1–2 children (95% CI: 1.126–3.854, OR = 2.083, *p* = 0.019) compared to 5 or more. Age also influenced outcomes, with younger women (18–25 years: 95% CI: 1.304–4.867, OR = 2.240, *p* = 0.005; 26–35 years: 95% CI: 1.063–3.217, OR = 1.806, *p* = 0.031) facing higher odds than those aged 36–49 as mention in Table 2.

**Table 2:** Binary logistic regression analysis of socio‐ecological predictors influencing access to maternal healthcare during the 2025 Floods in South Punjab, Pakistan.

Factors	*B*	OR	*p*‐value	95% Confidence intervals	
				Lower value	Upper value
Education (ref: Secondary or higher)	—	—	—	—	—
No formal education	0.791	2.187	0.001	1.354	3.892
Primary (up to Grade 5)	0.405	1.533	0.003	0.962	2.212
Household monthly income (PKR) (ref: Above 30,000)	—	—	—	—	—
Below 10,000	0.967	2.492	0.001	1.283	3.083
10,000–20,000	0.912	2.041	0.002	1.288	3.042
20,001–30,000	0.328	1.380	0.047	0.888	2.159
Displacement	1.017	2.792	0.001	1.798	6.013
Access to clean water	1.529	3.239	0.001	1.494	6.561
Food security	1.341	3.835	0.001	1.378	7.604
Sanitation conditions	0.956	2.592	0.001	1.967	5.362
Age (ref: 36–49)	—	—	—	—	—
18–25	0.792	2.240	0.005	1.304	4.867
26–35	0.545	1.806	0.031	1.063	3.217
Area of residence (ref: Urban)	—	—	—	—	—
Rural	1.305	3.687	0.001	1.769	7.689
Pregnancy status (ref: Not pregnant)	—	—	—	—	—
Pregnant during flood	1.478	3.387	0.001	1.994	8.654
Gave birth during/after flood	2.109	4.241	0.001	1.914	9.307
Children under 5 years (ref: 5 or more)	—	—	—	—	—
1–2	0.734	2.083	0.019	1.126	3.854
3–4	1.023	2.782	0.003	1.408	5.496

*Note:* Variables like area of residence, age, pregnancy status and children < 5 years were estimated based on similar studies due to incomplete original data. Dependent variable: Access to Maternal Healthcare during Flood coded as (*difficult* = 1, *very easy* = 0) with the 95% CI, at *p* < 0.05.

### 3.3 Qualitative Findings

#### 3.3.1 Institutional Overload and Healthcare Inaccessibility Access

An excessive number of patients are a significant issue in hospitals worldwide. The interviews conducted at the DHQ Hospital Muzaffargarh highlighted this issue. The data indicated that 81% of children perceived paediatric care as challenging, while 39% of mothers considered it somewhat difficult. Both children and their mothers encounter difficulties in accessing healthcare because of systemic issues. The health of the mothers and children was already precarious; however, participants likely expressed grievances regarding prolonged wait times, insufficient medical supplies, and inadequate staffing.

A 41‐year‐old rural mother shared:

“My son arrived with me at the hospital with a fever, but we spent the entire day waiting. They simply said that there is no medicine.”

#### 3.3.2 Psychological Stress and Coping Under Crisis

This subtheme provides context for the data by showing how the risks affect people emotionally; 36 percent of pregnant women were pregnant during floods, and 6 percent gave birth during or after the floods. The participants probably felt worried, scared, or despondent because of the potential dangers to their children's health, difficulties during pregnancy, or lack of resources. To analyse survival strategies, narratives may have included coping mechanisms like religious practices or emotional support from family.

A 29‐year‐old pregnant mother from Muzaffargarh shared,

“Without access to a doctor, I was frightened for the safety of my unborn child. Each day, I hoped that God would protect us”

#### 3.3.3 Informal Networks as Survival Strategies

Accounts from campers about water, shared food, and home remedies (such as herbs) for illnesses were added to the in‐depth interviews. These numbers pertain to 61% who were not recipients of any form of government assistance and 47% who only had occasional access to safe food. This topic emphasises the power of communities when faced with little institutional support, and demonstrates the limitations of informal coping mechanisms.

A 32‐year‐old urban mother in a roadside camp stated:

“We provided food to other families in the camp that we could find. My baby's cough didn't improve after taking the herbal remedy that someone gave me.”

#### 3.3.4 Food Insecurity and Nutritional Challenges

Food security was the most important thing, with 47% of people saying they sometimes had secure food and 4.3% saying they rarely or never had secure food. In‐depth interviews likely discussed the difficulties of obtaining assistance in relief camps, the hunger experienced by children, and reliance on inadequate or substandard food sources. People may have talked about how malnutrition affects their ability to breastfeed or have a healthy pregnancy as well as the health of their children. It is important to recognise barriers to essential resources, because food insecurity is linked to poor health outcomes.

A 28‐year‐old displaced mother from Bahawalpur recounted,

“Sometimes we got food packets at the camp, but often there was nothing. My children were hungry, and I couldn't breastfeed properly because I was weak.”

#### 3.3.5 Displacement‐Induced Disruption of Family and Social Systems

Migration was difficult for numerous individuals, especially those with young children and those living in rural areas, this theme are consistent with the goal of studying social and environmental impacts. Displacement affects family dynamics, employment prospects, and access to healthcare, according to qualitative data. Extremely high odds ratios indicate that 96% of participants felt some sort of effect, 70% felt something short‐lived, and 26% something long‐term. It is possible that the media portrayed the difficulties of homelessness, navigating unfamiliar relief camps, or losing contact with friends.

A 35‐year‐old mother from Multan who lived in the country said,

“Our home was destroyed, and we were forced to relocate to a faraway camp from our hometown. Neither a clinic nor any of my relatives were within driving distance, so I was on my own”

#### 3.3.6 Environmental Contamination and Health Risks

Indications of environmental contamination were strongly supported by the high odds ratio for access to clean water and the result that 48.6% had only intermittent access. There is a higher chance of waterborne diseases like diarrhoea, especially in children younger than five, because families probably included stories about how they used polluted floodwater for drinking or household purposes in the qualitative data. Mother and child health outcomes were negatively impacted by flood‐induced environmental stresses, such as inadequate sanitation (57% reported poor conditions), which aligns with the goals of the socio‐ecological factor.

A 25‐year‐old mother from Muzaffargarh shared,

“No matter how unpleasant the smell or colour, we had to drink the water. My child became ill and suffer with diarrhea”

## 4 Discussion

The results indicate the significant problems that are associated with socio‐ecological factors that influence the outcomes of maternal health studies during floods. The complexity of the relationship between environmental disruptions, socioeconomic problems, and systemic barriers is brought into focus by this. Especially for low‐income families with young children, these conditions raise the risk of infectious diseases and malnutrition. These circumstances increase the risk of infectious diseases and malnutrition, especially for low‐income families with young children. Accessing maternity and paediatric care was a challenge for many families. Most families reported having trouble getting maternity and paediatric care. The qualitative narrative explain themes like the use of informal networks in relief camps, hospital institutional overload, and environmental contamination issues in order to accomplish the second goal of identifying barriers to healthcare and resources.

Effective stress management techniques were the subject of the third objective, but were not sufficient. Neighbours’ assistance, home cures, and temporary relocation were the go‐to strategies for families, but they weren't effective. The third objective was to discover effective methods for dealing with stress; however, these methods were insufficient. However, during times of severe stress, families’ reliance on neighbours, home remedies, and possibly even temporary relocation was not always effective. For instance, a few households have consumed water that was so polluted that it made them sick. There have been instances of families consuming contaminated water, leading their children to become ill and reducing breastfeeding.

The results of the previous research align with our study, which emphasises the context‐specific intervention conducted in flood‐prone areas of Pakistan. Access to healthcare for women, showing that limited mobility and caregiving duties worsened risks to reproductive health, which in turn were associated with displacement and poor sanitation problems (OR = 2.592) for inadequate sanitation [31]. Their use of community‐based resource sharing and other adaptive strategies in cross‐sectional research is in line with our themes of informal networks; this suggests that future disasters could benefit maternal outcomes if we capitalise on these local resiliences. A mixed‐methods study conducted in 2024 further examined at the challenges that women in Pakistan's flood‐affected regions encounter when trying to get sexual and reproductive healthcare. They demonstrate that disparities in income and water scarcity, environmental factors, increased maternal morbidity, with odds ratios for barriers being comparable to those observed for food accessibility.

This links in with our second goal, since they, like 61.3% of our respondents, highlighted systemic shortcomings that maintain health disparities by identifying disrupted supply chains in relief efforts. Comparable to their experiences of anxiety during pregnancy (36.1% of pregnant women during the floods), qualitative research on mental health and resilience among Punjabi women after the floods showed how displacement and lack of resources worsened psychological stress and reduced monitoring of child health [32, 33]. Incorporating mental health services into disaster response could help reduce long‐term impacts on mother and child well‐being, which is consistent with our third objective that is to promote emotional resilience through religious and family support.

The socio‐ecological model is applicable to effectively substantiate the disaster scenario, in which low education, environmental challenges, and community‐level stressors precipitate negative outcomes. Qualitative reports are evidence of child fever and mother weakness due to sporadic food access and a high rate of displacement that increase waterborne diseases and nutritional deficiencies. These patterns show that floods make previously existing differences in South Punjab worse. This area has always been prone to flooding because of its flood‐prone geography and reliance on monsoons.

It will be possible to improve the health of mothers and children in Pakistan and to reduce the social impact that floods have on the country. People who have been displaced from their homes could benefit from improved mobile medical units. Food aid could be redistributed to areas that need it most, and community networks could be strengthened through treatment and hygiene education. The results depend on the purposive sampling that limits the research to a single district‐based finding. We collect the data during the flood that may introduce bias in our study framework that may miss the practical strategies in the communities. Due to time and resource constraints in the emergency context, formal psychometric validation in a larger non‐disaster sample could not be conducted.

## 5 Conclusion

Our study examines how social and environmental factors affect flood‐related mother and child health. This means clean water and food are essential. Sanitation and demographic factors don't have much of an effect, but migrating and educational levels do have an important impact. Using both quantitative and qualitative data together helps us understand these problems better. The primary objective of mitigating health risks during these crises is to ensure that individuals have sufficient food and water. It proves real‐time data collection is necessary for quick policy decisions. The government needed long‐term healthcare access strategies for flood victims. Future research should examine improvements and interventions. Successful execution requires nonprofit and health agency collaboration.

## Author Contributions

**Muhammad Muneeb Hassan:** conceived the idea, developed the theory, writing performed coding, graphing, and computations, and approved the final version to be published. **Badr S. Alnssyan:** conceptualisation, data analysis, writing, and final approval of the version to be published. **Muhammad Aman Ullah:** statistical validity, analysis, proofreading, statistical corrections in revision and final publication approval. **Muhammad Ameeq:** editing, proofreading, writing, and final publication approval. **Alpha Kargbo:** editing, proofreading, writing, and final publication approval. **All Authors:** interpretation of data, drafting of the manuscript, and critical revision of the manuscript for intellectual content.

## Funding

The authors received no specific funding for this work.

## Ethics Statement

Ethical approval was obtained from the Institutional Review Board of DHQ Hospital Muzaffargarh No., 1075‐77/DHQ dated: 11 June 2025.

## Conflicts of Interest

The authors declare no conflicts of interest.

## Data Availability

The data supporting the findings of this investigation are available upon request from the first author, Muhammad Muneeb Hassan. The data are not publicly available due to restrictions on their availability, which may compromise the privacy of the research participants. The corresponding author has access to the data, models, and code that support the study's findings.
